# Methanogen Levels Are Significantly Associated with Fecal Microbiota Composition and Alpha Diversity in Healthy Adults and Irritable Bowel Syndrome Patients

**DOI:** 10.1128/spectrum.01653-22

**Published:** 2022-11-02

**Authors:** Taojun Wang, Leander van Dijk, Iris Rijnaarts, Gerben D. A. Hermes, Nicole M. de Roos, Ben J. M. Witteman, Nicole J. W. de Wit, Coen Govers, Hauke Smidt, Erwin G. Zoetendal

**Affiliations:** a Laboratory of Microbiology, Wageningen University and Research, Wageningen, the Netherlands; b Division of Human Nutrition and Health, Wageningen University and Research, Wageningen, the Netherlands; c Wageningen Food and Biobased Research, Wageningen University and Research, Wageningen, the Netherlands; d Department of Gastroenterology and Hepatology, Hospital Gelderse Vallei, Ede, the Netherlands; e Cell Biology and Immunology, Wageningen University and Research, Wageningen, the Netherlands; University of Nebraska—Lincoln

**Keywords:** hydrogenotrophic functional groups, qPCR, fecal microbiota composition, sulfate-reducing bacteria, acetogens

## Abstract

Hydrogenotrophic microbes, primarily including the three functional groups methanogens, sulfate-reducing bacteria, and reductive acetogens, use hydrogen as an energy source and play an important role in maintaining the hydrogen balance in gut ecosystems. A distorted hydrogen balance has been associated with irritable bowel syndrome (IBS). However, the role of hydrogenotrophic microbes in overall microbiota composition and function remains largely unknown. This study aims to assess the distribution and stability of hydrogenotrophic functional groups in healthy adults (HAs) and IBS patients and their association with overall microbiota composition and IBS symptoms. A two-time-point study with 4 weeks in between was performed with 27 HAs and 55 IBS patients included. Our observations revealed that methanogens showed a bimodal distribution across samples. A high-level methanogen microbiota was consistently associated with higher alpha diversity, and its composition was significantly different from that of individuals with a low-level methanogen microbiota. In general, these associations were more pronounced in IBS patients than in HAs. The differences in the copy numbers of genes indicative of total bacteria and acetogens between HAs and IBS patients and their correlations with IBS symptom severity, anxiety, depression, and quality of life (QoL) were sampling time dependent. Hydrogenotrophic functional groups did not show negative abundance correlations with each other in HAs and IBS patients. These findings suggest that methanogen levels in the gut have a pronounced association with microbiota alpha diversity and composition, and the interactions between hydrogenotrophic functional groups are complex in gut ecosystems.

**IMPORTANCE** Hydrogenotrophic microbes play an essential role in the disposal of hydrogen and the maintenance of the hydrogen balance in gut ecosystems. Their abundances vary between individuals and have been reported to be associated with human gut disorders such as irritable bowel disease. This study confirms that methanogen levels show a bimodal distribution. Moreover, a high-level methanogen microbiota was associated with higher alpha diversity, and its composition was different from that of individuals with a low-level methanogen microbiota. These associations are more pronounced in IBS patients than in healthy subjects. In addition, associations between hydrogenotrophic microbes and IBS symptom scores vary over time, which argues for the use of longitudinal study designs. Last but not least, this study suggests that the different hydrogenotrophic microbes coexist with each other and do not necessarily compete for hydrogen in the gut. The findings in this study highlight the impact of methanogens on overall microbiota composition and function.

## INTRODUCTION

The human gut microbiota, comprising hundreds to thousands of microbial species, ferments dietary fibers that escape digestion and absorption, which results in the production of metabolites such as short-chain fatty acids, carbon dioxide, and hydrogen (1–3). Hydrogen is one of the main gases produced by hydrogen-producing microbes (hydrogenogens), with an estimated daily production of more than 13 liters in people consuming a typical Western diet (4). Hydrogen accumulation leads to a high partial pressure that inhibits the regeneration of the coenzyme NAD^+^ from NADH and thermodynamically restricts further microbial fermentation and growth (5, 6). The critical hydrogen disposal during bacterial fermentation is done via hydrogen-consuming microbes (hydrogenotrophs) (4), which decrease the partial pressure of hydrogen and maintain the hydrogen metabolism balance in gut ecosystems (7). The collection of hydrogenotrophic microbes in the human gut, collectively called the hydrogenotrophic microbiota, consists of three major functional groups, namely, methanogens that use hydrogen and carbon dioxide, producing methane; reductive acetogens that use hydrogen and carbon dioxide, producing acetate; and sulfate-reducing bacteria (SRB) that use hydrogen and sulfate, producing hydrogen sulfide (4, 6).

Irritable bowel syndrome (IBS) is the most commonly diagnosed functional gastrointestinal disorder, with an estimated 11% of people being affected globally (8). IBS is characterized by symptoms of abdominal pain typically accompanied by bloating and alterations in the stool pattern (9), which reduces quality of life (QoL) and work productivity and increases health care costs (8). Accumulating hydrogen is considered to be associated with IBS symptoms of abdominal pain and bloating, and a high hydrogen concentration in exhaled breath has been frequently observed in IBS patients compared to healthy adults (HAs) (6, 10, 11). Decreased colonic motility has been associated with higher methane concentrations and methanogen counts in both HAs and IBS patients, although it has to be mentioned that these observations have not consistently been reported (12–14). Higher SRB numbers have been found in constipated IBS patients than in HAs (12), and hydrogen sulfide produced by SRB has been linked to the modulation of pain-related signals, implying a role in hydrogen sulfide-mediated abdominal pain (15). Overall, hydrogenotrophic microbes have frequently been suggested to play a role in IBS (5, 6). However, these studies mainly compared hydrogenotrophic functional groups between HAs and IBS patients, the association of hydrogenotrophic functional groups with the overall microbiota composition, and IBS symptoms such as severity, depression, anxiety, and QoL, and their relationships with each other remain largely unknown. Moreover, the stability of hydrogenotrophic functional groups and their roles in HAs and IBS patients have not been investigated.

Therefore, we performed a two-time-point study over a period of 4 weeks, aiming to assess the distribution and stability of hydrogenotrophic functional groups in HAs and IBS patients and their association with overall microbiota composition and IBS symptoms such as symptom severity, depression, anxiety, and QoL over time.

## RESULTS

Twenty-seven HAs and 55 IBS patients were compared at two time points. The age, gender, and body mass index (BMI) of HAs and IBS patients were matched and thus were not significantly different (Table 1). Baseline characteristics significantly differed between HAs and IBS patients for symptom severity, quality of life, anxiety, and depression. No significant differences in the Bristol stool scale scores were observed between HAs and IBS patients. IBS patients had a lower intake of lactose and maltose, a higher trend toward glucose intake, and a lower trend toward protein intake than HAs (see Table S1 in the supplemental material).

**TABLE 1:** Baseline characteristics of the study population with 55 IBS patients and 27 healthy adults^*a*^

Parameter	Value for group		*P* value
	IBS (*n* = 55)	HAs (*n* = 27)	
Median age (yrs) (interquartile range)	42.0 (26.0–52.5)	35.0 (22.5–38.4)	0.590
No. (%) of male subjects	9 (16.4)	3 (11.1)	0.764
Mean BMI (kg/m^2^) ± SD	22.8 ± 2.8	23.3 ± 3.0	0.508
Median IBS-SSS (interquartile range)	140 (100–20)	60 (30–90)	0.000
Median Bristol stool scale score (interquartile range)	3 (3–4)	4 (3–6)	0.270
Median IBS-QoL score (interquartile range)	75.7 (57.4–85.3)	99.3 (98.9–100.0)	0.000
Median anxiety score (interquartile range)	7.0 (4.0–11.5)	4.0 (3.0–6.0)	0.003
Median depression score (interquartile range)	2.0 (1.0–5.5)	(0.5–2.5)	0.025

a Data are presented as means ± standard deviations or medians (interquartile ranges) when skewed. Age, IBS symptom severity score (IBS-SSS), Bristol stool scale score, IBS quality of life (IBS-QoL) score, anxiety score, and depression score were tested with the Mann-Whitney U test. Body mass index (BMI) was tested with an independent-sample *t* test. Gender was tested with Pearson’s chi-square test.

### Differences in total bacteria and acetogens between healthy adults and IBS patients are sampling time dependent.

**FIG 1:**
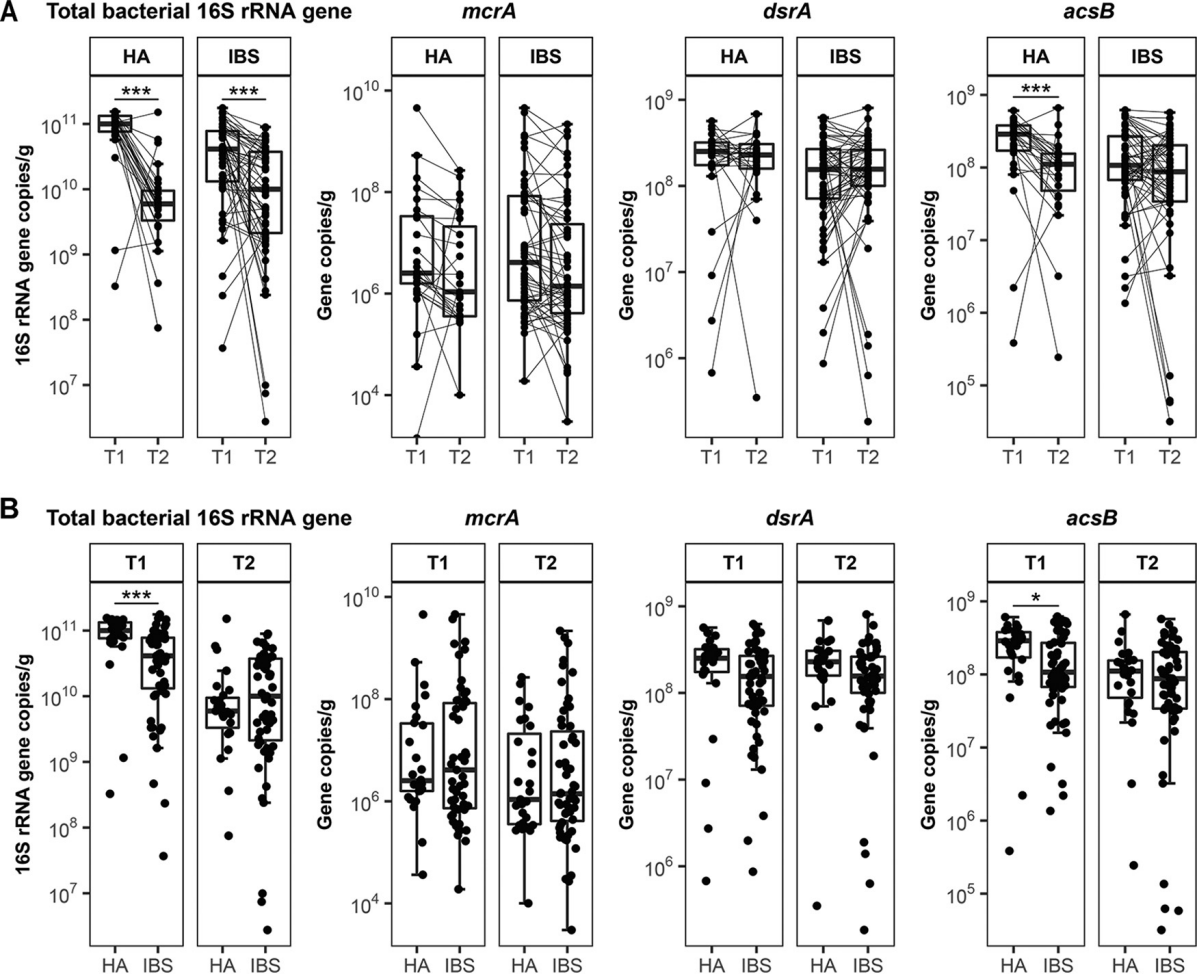
Box plots showing the stability of total bacterial 16S rRNA gene counts and hydrogenotrophic functional groups (*mcrA*, methanogens; *dsrA*, sulfate-reducing bacteria; *acsB*, acetogens) over time (A) and time-point-specific comparisons between healthy adults (HA) and IBS patients (B). Samples taken at different time points are connected by solid lines per subject. Differences between groups were tested with the Mann-Whitney U test. *, *P* < 0.05; **, *P* < 0.01; ***, *P* < 0.001.

Differences in the copy numbers of genes representative of total bacteria and hydrogenotrophic functional groups within individuals were observed over time (Fig. 1A). Total bacterial 16S rRNA gene copies significantly decreased over time in both HAs and IBS patients. A much lower *acsB* copy number was observed at time point 2 (*T*_2_) than at *T*_1_ in HAs. In contrast, *acsB* copy numbers remained stable over time in IBS patients. There were not any significant differences observed for *dsrA* or *mcrA* between time points in either HAs or IBS patients. When comparing gene copy numbers between HAs and IBS patients at each time point, we observed higher copy numbers of total bacterial 16S rRNA genes and *acsB* in HAs at *T*_1_, while these differences were not observed at *T*_2_ (Fig. 1B). For copy numbers of *mcrA* and *dsrA*, no differences were found between HAs and IBS patients at both time points. The differences in total bacteria and hydrogenotrophic functional groups between HAs and IBS patients were not associated with diet. Stratification of IBS patients into subgroups based on the predominant stool patterns demonstrated that only total bacteria and *acsB* were significantly lower in constipation-predominant IBS than in HAs at *T*_1_ (Table S2 and Fig. S1).

### Correlations of total bacteria and acetogens with IBS symptoms are sampling time dependent.

**FIG 2:**
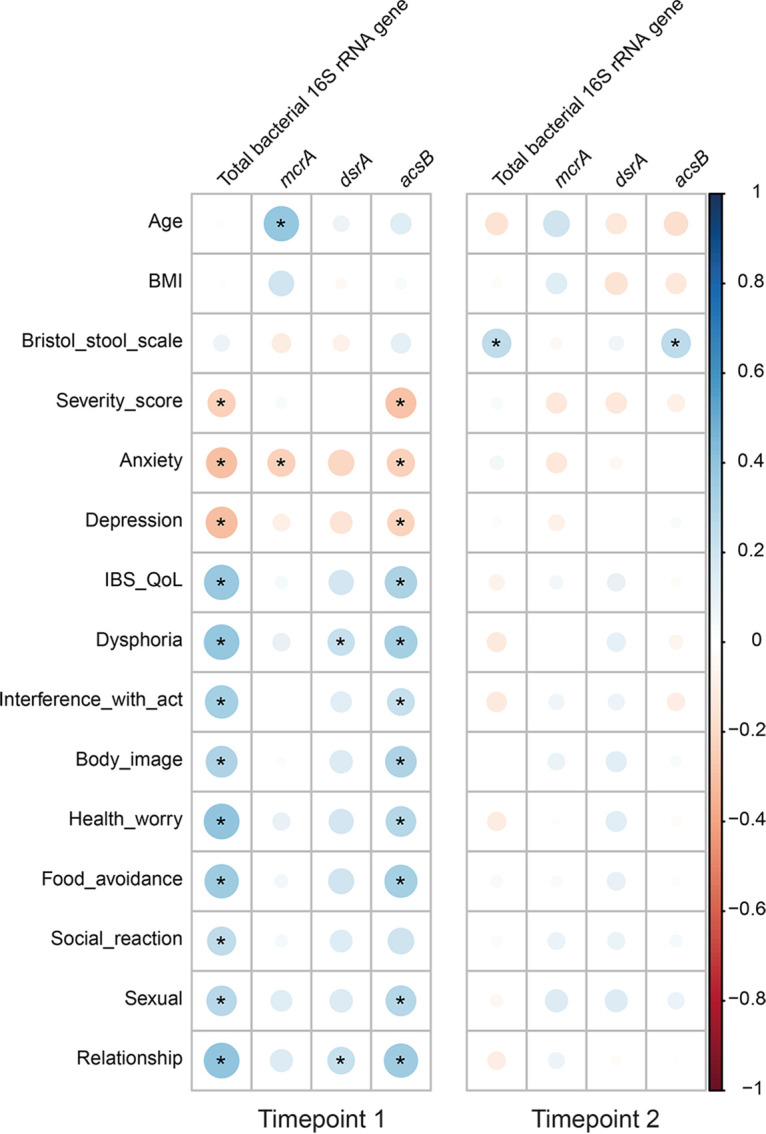
Spearman’s correlation analyses of copy numbers of total bacterial 16S rRNA genes and functional genes representative of the different hydrogenotrophic functional groups (*mcrA*, methanogens; *dsrA*, sulfate-reducing bacteria; *acsB*, acetogens) with population characteristics over time. This analysis includes 27 healthy adults and 55 IBS patients. Significant correlations (*P* < 0.05) are indicated with an asterisk. Abbreviations: BMI, body mass index; IBS-QoL, IBS quality of life.

The demographic data and symptoms measured in HAs and IBS patients did not change during the study, except for the IBS quality of life (IBS-QoL) subindices dysphoria, body image, and relationships for IBS patients (Table S3). Spearman’s correlations of the IBS symptoms with the copy numbers of total bacterial 16S rRNA genes and genes indicative of the different hydrogenotrophic functional groups indicated that total bacterial 16S rRNA genes and *acsB* were negatively correlated with the IBS symptom severity score (IBS-SSS), anxiety, and depression but positively correlated with IBS-QoL and its subindices at *T*_1_ (Fig. 2). However, we did not observe any of these correlations at *T*_2_. For the demographic data, only age was positively correlated with *mcrA* at *T*_1_, and the Bristol stool scale score was positively correlated with counts of total bacterial 16S rRNA genes and *acsB* at *T*_2_. However, when only IBS patients were included in the correlation analyses, *mcrA* and *acsB* were positively correlated with age and the Bristol stool scale score, respectively, at both time points (Fig. S2).

### Methanogen levels are significantly associated with fecal microbiota alpha diversity and composition.

**FIG 3:**
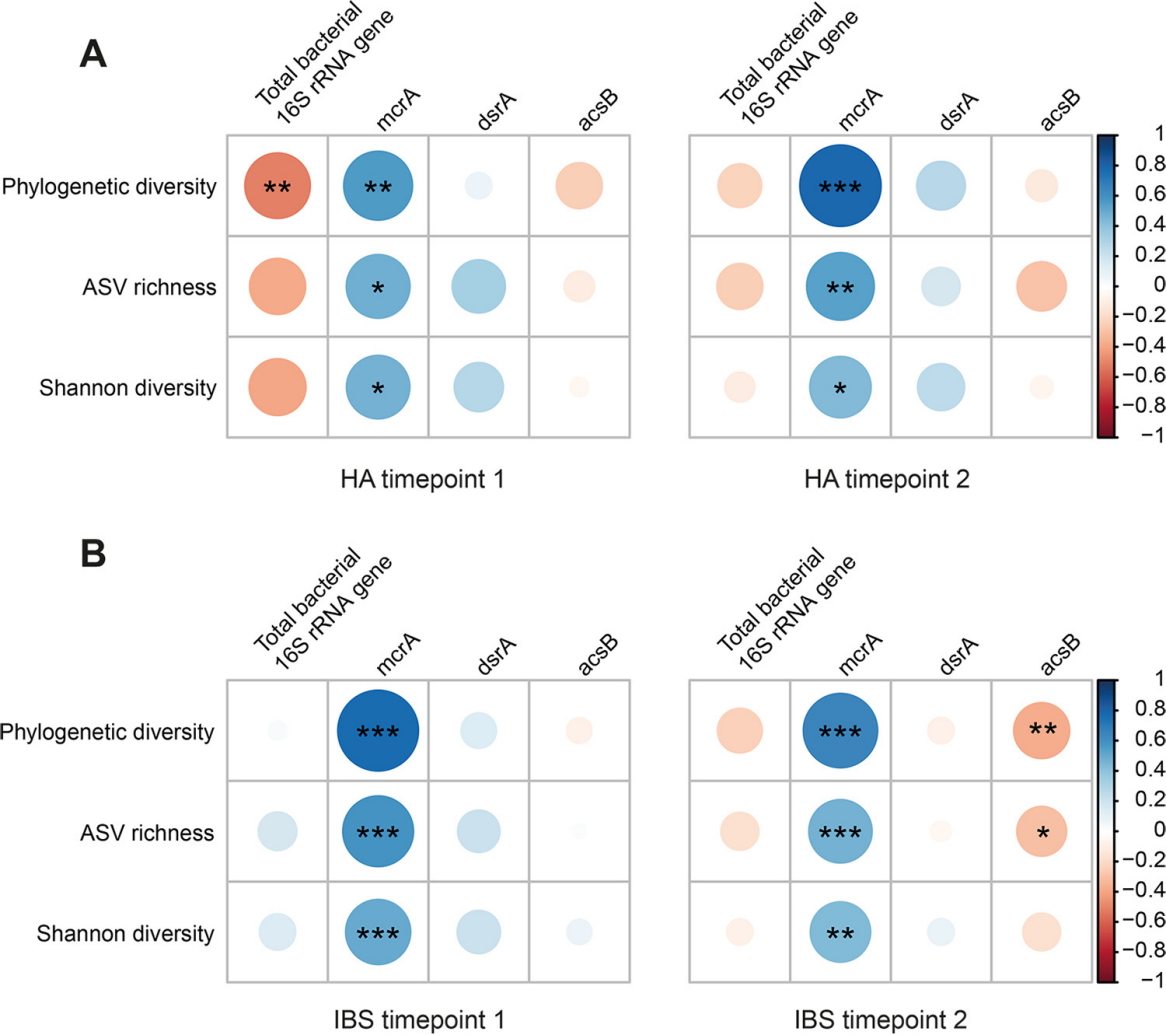
Spearman’s correlation between hydrogenotrophic functional groups (*mcrA*, methanogens; *dsrA*, sulfate-reducing bacteria; *acsB*, acetogens) and the microbial alpha diversity metrics phylogenetic diversity, ASV richness, and Shannon diversity in healthy adults (HA) (A) and IBS patients (B) over time. *P* values are highlighted with different asterisks (*, *P* < 0.05; **, *P* < 0.01; ***, *P* < 0.001).

Spearman correlation analysis was performed between alpha diversity metrics (phylogenetic diversity, amplicon sequence variant [ASV] richness, and Shannon diversity) and total bacteria and hydrogenotrophic functional groups in both HAs and IBS patients at both time points (Fig. 3). Remarkably, *mcrA* was positively correlated with phylogenetic diversity, ASV richness, and Shannon diversity in both HAs and IBS patients at both time points. In contrast, no significant correlation was observed between fecal microbiota alpha diversity indices and *dsrA*. As for *acsB*, a negative correlation with phylogenetic diversity and ASV richness was observed at *T*_2_ in IBS patients; however, this was not observed at *T*_1_.

The abundance of the methanogenic genus *Methanobrevibacter* in the human gut shows a bimodal distribution, which means that this genus is either very abundant or nearly absent (16). Despite the limited number of measurements in this study, the abundance distribution of *mcrA* showed two peaks in both HAs and IBS patients, with a cutoff for the two peaks at ~10^7^ gene copies/g. This confirmed that methanogens had a bimodal distribution in our study populations (Fig. S3). In contrast, this bimodality was not observed with the functional genes indicative of SRB or acetogens. To investigate how methanogens were associated with the overall microbiota composition, subjects were stratified into high-level methanogen (HM) and low-level methanogen (LM) groups based on the number of *mcrA* gene copies. The ratio of subjects with HM to subjects with LM did not differ over time. In addition, no difference in the HM/LM ratio was found between HAs and IBS patients at both time points (Table S4). As expected, this stratification resulted in a significant difference in the abundances of *mcrA* between HM and LM (Fig. S4). In contrast, no significant abundance differences were observed for total bacterial 16S rRNA genes or the genes indicative of SRB and acetogens.

**FIG 4:**
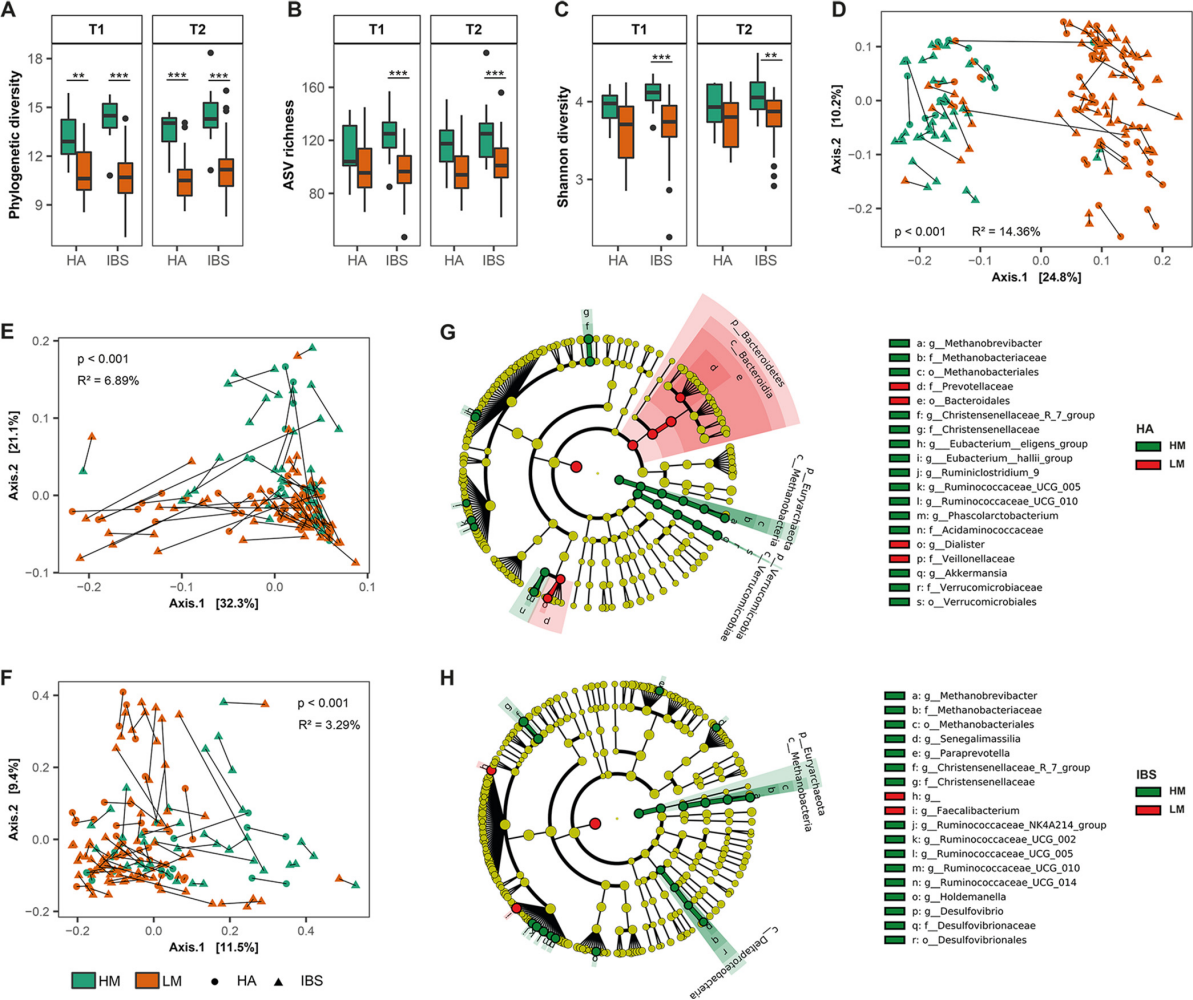
Stratification of subjects based on methanogen levels shows a distinct microbiota composition associated with high-level methanogens (HM). (A to C) Significance was observed in phylogenetic diversity (A), ASV richness (B), and Shannon diversity (C) between HM and low-level methanogens (LM) in IBS patients at both time points. A Mann-Whitney U test was used to test for significance. (D to F) PCoA of microbiota composition was carried out based on unweighted (D) and weighted (E) UniFrac distances and Bray-Curtis dissimilarity (F). PERMANOVA showed that the microbiota composition of HM is different from that of LM in IBS patients at both time points. Samples taken at different time points are connected by solid lines per subject. (G and H) Cladograms representing the genera that were significantly different between HM and LM in healthy adults (G) and IBS patients (H) using the linear discriminant analysis effect size (LEfSe) method. HM and LM were assigned as class. Time point 1 and time point 2 were assigned as subclass. Taxa or nodes highlighted in red and green were significantly more abundant in LM and HM, respectively. The nomenclature of microbial genus-level taxa is based on the highest achievable taxonomic resolution at the phylum, class, order, family, or genus level. *, *P* < 0.05; **, *P* < 0.01; ***, *P* < 0.001.

Interestingly, the stratification of subjects based on methanogen levels indicated a significant association with microbiota alpha diversity indices and microbiota composition (Fig. 4). Both HAs and IBS patients with HM had higher phylogenetic diversity (Fig. 4) than those with LM at both time points, which is expected because methanogens as an independent domain (archaea) play an important role in determining the phylogenetic distance within the algorithm. Nevertheless, even without considering the phylogenetic distance, ASV richness (Fig. 4B) and Shannon diversity (Fig. 4C) were significantly higher in the IBS patients with HM than in those with LM at both time points as well. In contrast, for ASV richness and Shannon diversity, we did not observe a significant difference between HM and LM in HAs. In addition, permutational multivariate analysis of variance (PERMANOVA) revealed significant differences between the fecal microbiota of subjects with HM and that of subjects with LM based on unweighted (Fig. 4D) and weighted (Fig. 4E) UniFrac distances as well as Bray-Curtis dissimilarity (Fig. 4F), with samples from both HAs and IBS patients included. As could be expected, a larger effect based on UniFrac distances that take phylogeny into account was observed for differences between HM and LM. When HAs and IBS patients were analyzed separately, however, significant differences between HM and LM based on Bray-Curtis dissimilarity were found only for IBS patients at both time points (Fig. S5), whereas this was observed only at *T*_2_ in HAs, indicating that methanogen levels have a stronger association with the microbiota composition in IBS patients than in HAs. To further determine in more detail which taxa were associated with HM and LM, the linear discriminant analysis (LDA) effect size (LEfSe) algorithm was performed (Fig. 4G and H; Fig. S6). This indicated that besides methanogens, the family *Christensenellaceae* and the genera *Ruminococcaceae* UCG_005 and *Ruminococcaceae* UCG_010 were consistently associated with HM over time in both HAs and IBS patients. In HAs, the genera *Akkermansia*, *Phascolarctobacterium*, *Ruminiclostridium* 9, Eubacterium hallii group, and Eubacterium eligens group showed a higher relative abundance in HM, whereas the relative abundances of the phylum *Bacteroidetes* and the family *Veillonellaceae* were higher in LM. However, this was not observed in IBS patients.

### Hydrogenotrophic functional groups did not show negative abundance correlations with each other.

**FIG 5:**
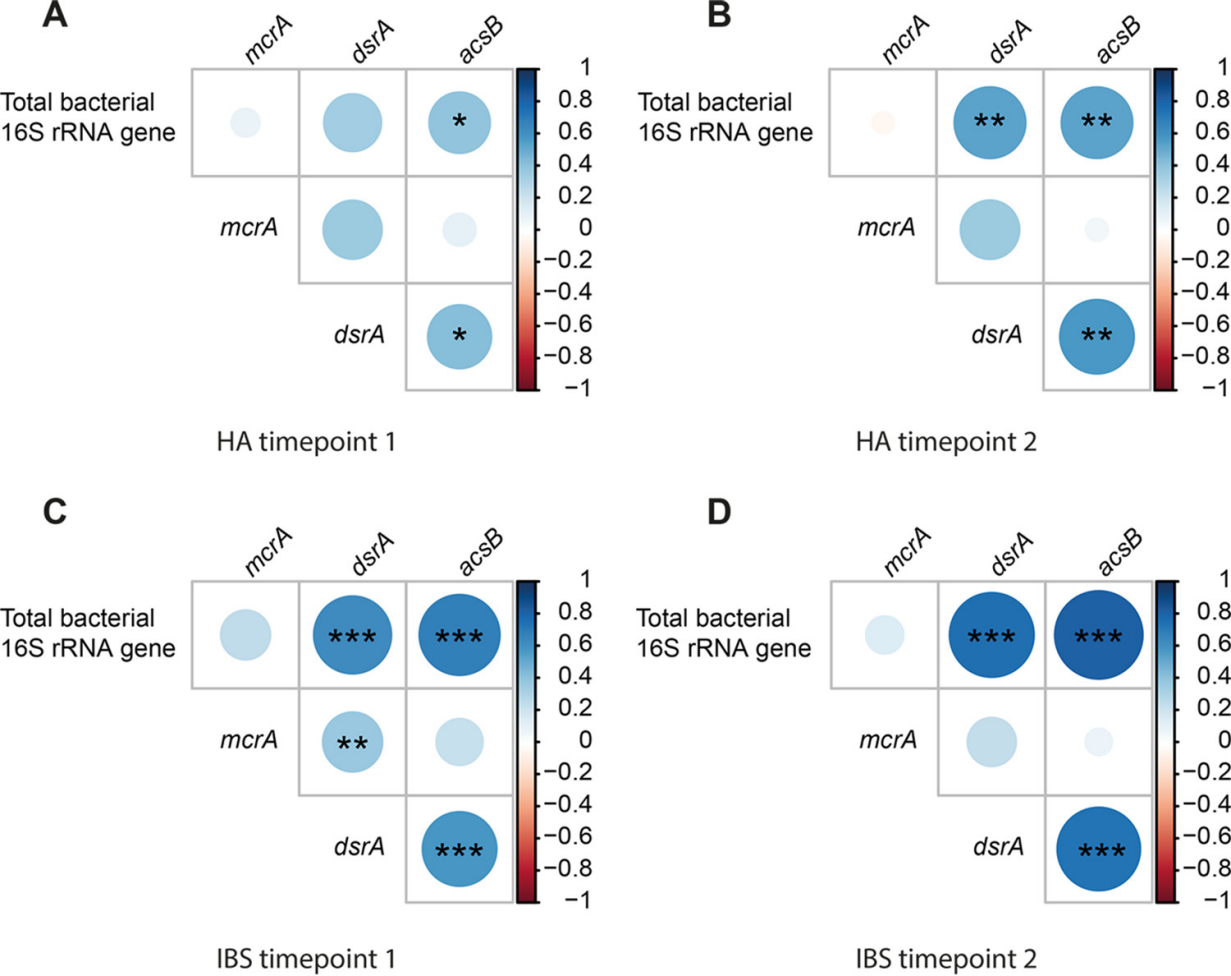
Correlation between total bacterial 16S rRNA genes and hydrogenotrophic functional groups (*mcrA*, methanogens; *dsrA*, sulfate-reducing bacteria; *acsB*, acetogens). Subjects are stratified into HAs (at *T*_1_ [A] and *T*_2_ [B]) and IBS (at *T*_1_ [C] and *T*_2_ [D]) at two time points. Statistical significance was determined using the Spearman method. *, *P* < 0.05; **, *P* < 0.01; ***, *P* < 0.001.

Interestingly, no negative correlations were found between the different hydrogenotrophic functional groups in either HAs or IBS patients over time (Fig. 5). Total bacterial 16S rRNA gene copy numbers were positively correlated with *acsB* at both time points in HAs and IBS patients. Total bacterial 16S rRNA genes and *dsrA* were positively correlated at both time points in IBS patients, while in HAs, a positive correlation was observed only at *T*_2_. No correlation was observed between the bacterial 16S rRNA gene and *mcrA*. As for the copy number of *acsB*, it was positively correlated with *dsrA* in both HAs and IBS patients at both time points. In contrast, *acsB* was not correlated with *mcrA*, and *mcrA* was not correlated with *dsrA* in HAs, while they were positively correlated in IBS patients at *T*_1_ but not at *T*_2_. Collectively, these findings indicate that the hydrogenotrophic functional groups coexist, and the fact that no negative correlations were observed suggests that these hydrogenotrophic functional groups may not compete with each other in the gut ecosystems of HAs and IBS patients.

## DISCUSSION

In this study, we compared the abundances of total bacteria and three different hydrogenotrophic functional groups, namely, methanogens, SRB, and acetogens, between HAs and IBS patients at two time points spanning a period of 4 weeks and showed an association of methanogen levels with microbiota alpha diversity and composition. Moreover, we investigated the correlation of hydrogenotrophic functional groups with each other and IBS symptoms such as severity, depression, anxiety, and QoL. We mainly found that total bacteria and acetogens showed differences between HAs and IBS patients and correlations with IBS symptoms such as the severity score, anxiety, depression, and QoL albeit depending on the time of sampling. The different hydrogenotrophic functional groups coexisted and were not found to be negatively correlated, suggesting that they may not compete in the human gut. Higher fecal methanogen levels were significantly associated with higher microbiota alpha diversity and showed compositional differences compared to low methanogen levels and more so in IBS patients than in HAs.

Cross-sectional studies have been commonly used to identify microbial signatures associated with IBS (17). Previously, no differences in total bacterial abundances were observed between IBS patients and healthy controls (18). However, using a two-time-point comparison in our study, the differences in total bacterial abundances between HAs and IBS patients varied over the short 4-week period included in this study. Besides total bacterial abundance, we also observed differences between time points in the abundances of hydrogenotrophic functional groups within individuals. In addition, we observed that the abundances of total bacteria and acetogens were correlated with IBS symptom severity and other IBS symptoms such as anxiety, depression, and QoL at *T*_1_, but this was not the case at *T*_2_, indicating that correlations at a single time point can be coincidental, as was shown previously (19). This highlights that snapshot cross-sectional comparisons or correlations may not reliably identify microbial signatures in chronic diseases without a longitudinal sampling process.

It has been considered that different hydrogenotrophic functional groups may compete because all of them use hydrogen as an energy source (4, 5). To support this, a mutually exclusive relationship between methanogens and SRB has been suggested. SRB are rarely detected in the gut microbiota of methane excretors that have a significantly higher number of methanogens than non-methane excretors, while the gut microbiota of non-methane excretors harbors higher numbers of SRB (20). However, other studies did not observe such mutual exclusivity, and no significant relationship was found between methanogens and SRB (21, 22). To this end, our study did not show negative abundance correlations among any of the three hydrogenotrophic functional groups over time in either HAs or IBS patients. An explanation as to why they coexist could be that the hydrogen supply is not a limiting factor or that hydrogen is considered essential to methanogens, whereas SRB and reductive acetogens are more metabolically flexible. SRB can also use other compounds such as lactate in the absence of hydrogen and thereby become hydrogen producers (23, 24). Reductive acetogens are not restricted to hydrogen as an energy source either and instead are also able to ferment carbohydrates (25, 26). Considering the complexity of gut ecosystems, including the nutrient supply, variable environmental conditions throughout the gut, as well as the metabolic flexibility of some of the hydrogenotrophic microbes, the interactions between hydrogenotrophic microbes in the gut are difficult to decipher (5). Therefore, insights into the interactions among the three hydrogenotrophic functional groups are needed and can be obtained, for example, by coculturing *in vitro* or cocolonization *in vivo*.

It has been frequently reported that the level of hydrogen production is higher in IBS patients than in healthy controls, although this is not consistent between studies, which may suggest that bacterial fermentation may be an important factor in IBS pathogenesis (11, 27, 28). Interestingly, in this study, compared to HAs, methanogens were found to have a pronounced association with fecal microbiota alpha diversity and composition in IBS patients. Hence, it remains speculative that higher-level hydrogen production increases the association of microbiota composition with methanogens in IBS, which requires further investigation.

*Methanobrevibacter* is the dominant methanogenic genus in the human gut ecosystem, using hydrogen and carbon dioxide for the production of methane. A bimodal distribution of *Methanobrevibacter* has been reported (16), which is in line with our finding that methanogens quantified via *mcrA* showed a bimodal distribution as well. In addition, based on detectable breath methane, people can be stratified into methane excretors, with a significantly higher number of methanogens (~10^9^ CFU/g), and non-methane excretors, with significantly lower methanogen counts (~10^4^ CFU/g), in the stool (4, 29, 30). Remarkably, in this study, stratifying subjects into HM and LM showed that subjects with HM harbored a fecal microbiota with high alpha diversity and a distinct microbiota composition compared to those of subjects with LM. This cannot be explained solely by the fact that phylogenetically distinct methanogenic archaea differed in prevalence and abundance but are also related to differences with respect to other bacterial taxa as this was also observed with algorithms that do not include phylogenetic distance. Hydrogen accumulation leads to higher hydrogen partial pressure that would inhibit the regeneration of the coenzyme NAD^+^ from NADH and thermodynamically restrict further microbial fermentation and growth (5, 6). Hydrogenotrophic microbes play an essential role in reducing the hydrogen partial pressure and facilitating the fermentation process. We speculate that methanogens contribute strongly to the hydrogen partial pressure and thereby facilitate the fermentation capacity for a diverse microbiota. This is supported by findings that cellulose-degrading bacterial communities in the human gut differ between individuals according to the presence or absence of methanogens (31). When looking at taxa associated with HM or LM, we observed that the family *Christensenellaceae* was consistently associated with HM in both HAs and IBS patients. Coculturing species of the *Christensenellaceae* and Methanobrevibacter smithii indicated a syntrophic relationship between *Christensenella* and *Methanobrevibacter* via interspecies hydrogen transfer (32). Hence, the consistent cooccurrence of both taxa observed in this study likely reflects their syntrophic interaction in the intestine with HM. In addition, greater metabolic efficiency of saccharolytic bacteria has been observed when these bacteria were cocultured with M. smithii *in vitro* (33) or cocolonized with M. smithii in a humanized mouse model (34). Overall, these observations indicate that the introduction of methanogens may have a significant impact on the overall microbiota composition and function.

In conclusion, an HM microbiota was consistently associated with higher alpha diversity, and its composition was significantly different from that of LM. Since we observed a consistent cooccurrence between M. smithii and *Christensenellaceae*, we speculate that syntrophic interactions between these taxa play an important role in the HM ecosystem. Fluctuations in the time of the abundance of hydrogenotrophic functional groups, IBS symptoms, as well as their correlations suggest that a longitudinal sampling process is necessary to identify a reliable microbial signature. The coexistence and nonnegative correlations between the abundances of different hydrogenotrophic functional groups as well as the different associations with microbiota diversity and composition in HAs and IBS patients imply that the interactions between hydrogenotrophic functional groups are complex and need further elucidation in gut ecosystems.

## MATERIALS AND METHODS

**FIG 6:**
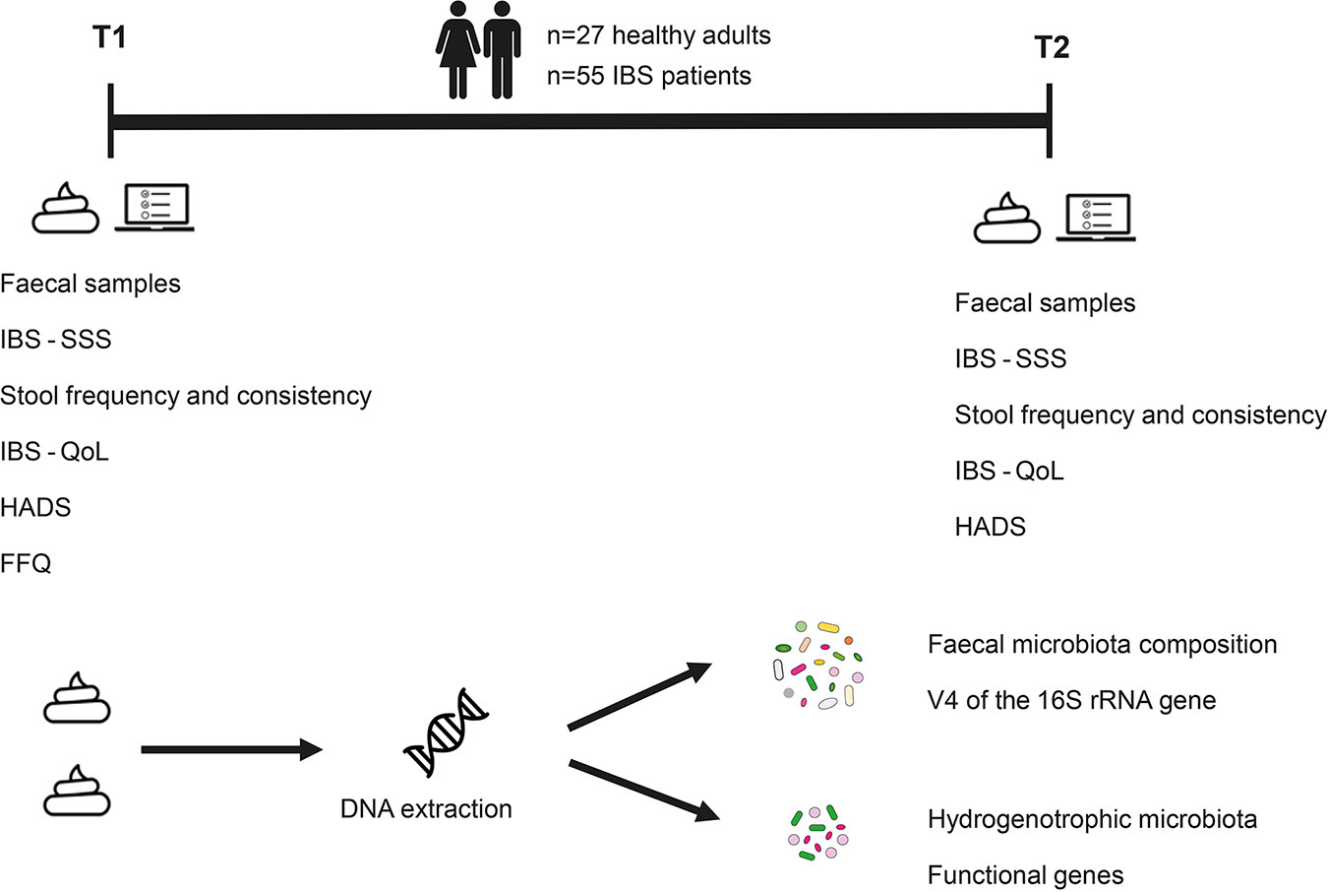
Schematic overview of the study design. Twenty-seven healthy adults and 55 irritable bowel syndrome (IBS) patients were included in this study. Fecal samples and questionnaire data were collected. The fecal microbiota composition was analyzed by sequencing the V4 region of the 16S rRNA gene, and three different groups of hydrogenotrophic microbes (methanogens, sulfate-reducing bacteria, and acetogens) were quantified by quantitative PCR targeting representative functional genes. SSS, symptom severity score; QoL, quality of life questionnaire; HADS, hospital anxiety and depression score; FFQ, food frequency questionnaire.

The details of the study design, subject recruitment, questionnaire data collection, and fecal microbiota composition profiling were described previously (35). Briefly, this was an observational study that included two time points (*T*_1_ and *T*_2_) with 4 weeks in between. Thirty HAs and 91 IBS patients who were matched for age, gender, and body mass index (BMI) were recruited for *T*_1_. Based on their symptom severity scores, the 30 IBS patients with the most severe symptoms and the 30 patients with the least severe symptoms were selected for *T*_2_. A subset of subjects who completed both time points (27 HAs and 55 IBS patients) was included in this study (Fig. 6).

### Study design.

The recruited subjects were aged 18 to 65 years and had a BMI of between 18.5 and 30.0 kg/m^2^. Of the 55 IBS patients, 11 fulfilled Rome IV criteria, 5 were diagnosed with IBS by a physician, and 39 had a physician’s diagnosis as well as the fulfillment of Rome IV criteria. The diagnosis of IBS was checked and confirmed for all participating IBS patients by the gastroenterologist on our research team (B. J. M. Witteman). HAs and IBS patients who had any other gastrointestinal or systemic diseases or antibiotic use less than 3 months before the start of the study or who were pregnant or breastfeeding were excluded. Questionnaires were completed by HAs and IBS patients at both time points for comparison. The validated IBS symptom severity score (IBS-SSS) was used to assess IBS severity (36). The 34-item IBS-QoL questionnaire was used to assess QoL, which gave a score for total IBS-QoL and the subscales dysphoria, interference with activity, body image, health worry, food avoidance, social reaction, sexual life, and relationships (37). The hospital anxiety and depression score questionnaire (38) was completed by subjects, with a score of ≥8 indicating substantial depressive or anxious symptoms (39). The seven types of the validated Bristol stool chart were used to assess the stool consistency as well as the stool frequency to have the predominant stool pattern determined by participants who ranked their stools during the week before sampling from most to least frequent (40). A semiquantitative 83-item food frequency questionnaire was used to assess the habitual dietary intake in the month preceding *T*_1_ (41, 42). The Dutch food composition table was used to calculate dietary intake (43). Subjects were instructed to keep their diet as similar as possible during the study period. Fecal samples were collected at both time points. The fecal microbiota composition was determined based on the V4 region sequences of the bacterial and archaeal 16S rRNA genes (Illumina HiSeq 2500, 150-bp paired end). Total bacteria, methanogens, SRB, and acetogens were quantified by quantitative PCR (qPCR).

### Total bacterial and hydrogenotrophic functional group profiling.

Total bacteria were quantified by determining the 16S rRNA gene copy number, while methanogens, SRB, and acetogens were quantified by determining the copy number of the functional genes *mcrA*, *dsrA*, and *acsB*, respectively (Table 2), which are representative of their respective metabolic pathways (4, 44). Standards for quantification included the amplified 16S rRNA gene of Escherichia coli JM109 for total bacteria and genomic DNA isolated from Desulfovibrio piger DSM 749, Blautia hydrogenotrophica DSM 10507, and Methanobrevibacter smithii DSM 861 for *dsrA*, *acsB*, and *mcrA*, respectively. Standards were serially 10-fold diluted with nuclease-free water. The sample DNA concentration was adjusted to 1 ng/μL with nuclease-free water for qPCR.

**TABLE 2:** qPCR assays in this study^*a*^

Group	Gene	Primers	Annealing temp (°C)	Standard	Reference
Total bacteria	16S rRNA	Uni331 F, Uni797 R	60	E. coli JM109	50
Methanogens	*mcrA*	qmcrA-F, qmcrA-R-d	60	M. smithii DSM 861	51
SRB	*dsrA*	DSR1-F, DSR-R	60	*D. piger* DSM 749	52
Acetogens	*acsB*	ACS_f, ACS_r	52	*B. hydrogenotrophica* DSM 10507	53

a SRB, sulfate-reducing bacteria; *mcrA*, subunit of methyl coenzyme M reductase genes; *dsrA*, subunit of dissimilatory (bi)sulfite reductase genes; *acsB*, subunit of acetyl-CoA synthase genes.

All qPCRs were carried out in triplicate with an iCycler iQ real-time detection system (Bio-Rad Laboratories BV). Each reaction mixture with a total volume of 10 μL contained 5 μL 2× iQ SYBR green (Bio-Rad Laboratories BV), 2 ng DNA template, forward and reverse primers, and nuclease-free water. For each group, the primers and annealing temperatures used are listed in Table 2.

### Fecal microbiota composition analysis.

The fecal microbiota composition was determined by sequencing the V4 region of the 16S rRNA gene, and NG-Tax 2.0 was used to process the raw sequence data for amplicon sequencing variant (ASV) picking with default settings and for taxonomic assignments as described previously (35). Alpha diversity (within-sample diversity) and beta diversity (between-sample diversity) were calculated at the ASV level using Phyloseq (45). For alpha diversity metrics, ASV richness, Shannon diversity, and phylogenetic diversity were calculated. To visualize beta diversity, principal-coordinate analysis (PCoA) based on unweighted (considering the presence/absence of ASVs) and weighted (considering ASVs and their relative abundances) UniFrac (46) distances and Bray-Curtis dissimilarity was performed. Subjects were stratified into high-level methanogens (HM) or low-level methanogen (LM) groups based on the bimodal distribution of *mcrA* copy numbers observed in our study (HM, >10^7^ *mcrA* copies/g; LM, ≤10^7^ *mcrA* copies/g). The linear discriminant analysis (LDA) effect size (LEfSe) algorithm was used to determine the differences in microbial genus-level taxa between LM and HM subjects (47).

### Statistical analysis.

Continuous data were presented as means ± standard deviations or medians and interquartile ranges when skewed. Categorical data were presented as counts and percentages. Differences between HAs and IBS patients or between HM and LM were tested by one-way analysis of variance (ANOVA) or a Mann-Whitney U test when not normally distributed. Differences in categorical data were assessed using Pearson’s chi-square test. The significances of the correlation between total bacteria and hydrogenotrophic functional groups, the correlation between hydrogenotrophic functional groups and questionnaire data, and the correlation between the functional genes and alpha diversity metrics were determined using Spearman’s rank correlation coefficient. All of the data were analyzed in R version 4.0.0 (48).

All *P* values for the multiple pairwise tests were corrected using the Benjamini-Hochberg false discovery rate (49). A *P* value (or corrected *P* value) of <0.05 was considered to indicate statistical significance, and a trend was considered when 0.05 ≤ *P* (or corrected *P*) < 0.1.

### Ethics.

This study was approved by the medical ethics committee of Wageningen (18/06) and conducted according to the Declaration of Helsinki. All participants signed written informed consent. The study was registered at ClinicalTrials.gov under identifier NCT03720314 on 25 October 2018.

### Data availability.

Microbial sequencing data have been deposited in the European Nucleotide Archive database under accession number PRJEB44533.
