# Supplementary material for: Methanogen Levels Are Significantly Associated with Fecal Microbiota Composition and Alpha Diversity in Healthy Adults and Irritable Bowel Syndrome Patients
Source: Microbiol Spectr. 2022 Nov 2;10(6):e01653-22. doi: 10.1128/spectrum.01653-22 (PMC9769613; doi:10.1128/spectrum.01653-22)
Supplement: Supplemental file 1 — Supplemental material. Download spectrum.01653-22-s0001.pdf, PDF file, 0.9 MB [file spectrum.01653-22-s0001.pdf]

Supplementary Material

Supplementary Table 1: Dietary intake of the study population

	IBS (n=55)	HA (n=27)	p values
Energy (kcal)	1895.0 (1626.9 - 2157.2)	2105.2 (1699.3 - 2449.7)	0.180
Protein (g)	67.8 (59.2 – 77.4)	77.4 (66.2 – 92.4)	**0.073**
Total fat (g)	76.6 (65.1 – 101.8)	82.7 (67.3 – 100.0)	0.590
Saturated fat (g)	26.8 (18.1-32.0)	31.2 (23.1-35.4)	0.200
Total Carbohydrates (g)	207.7 (181.3 – 244.0)	228.3 (177.8 – 269.8)	0.260
Glucose (g)	9.7 (7.1 – 12.9)	8.6 (6.0-10.8)	**0.059**
Fructose (g)	14.6 ± 6.0	12.8 ± 5.5	0.164
Lactose (g)	6.4 (2.2 – 12.3)	11.3 (7.7 – 19.2)	**0.011**
Maltose (g)	1.8 (1.2 – 2.6)	2.6 (2.1 – 3.6)	**0.004**
Saccharose (g)	26.2 (19.0 – 33.9)	24.0 (18.5 – 38.5)	0.690
Polysaccharides (g)	120.7 (96.1 – 135.8)	130.1 (106.9 – 152.0)	0.190
Dietary fiber (g)	24.0(20.34 – 31.27)	24.2 (21.6 – 30.4)	0.800
Alcohol (g)	2.3 (0.0 – 5.7)	3.3 (0.1 – 10.7)	0.270
Water (g)	2704.0 ± 705.9	2472.0 ± 668.1	0.154

Twenty-seven healthy adults and fifty-five IBS patients were included for the comparative analysis. Data are presented as mean ± standard deviation or median (interquartile range) when skewed. Differences between groups were determined with an independent sample t-test or Mann-Whitney U test when skewed. The definition of each nutrient is according to the Dutch food composition table. In line with the Dutch food composition table, dietary fibre is not included in the calculation of total carbohydrates but treated as a separate category. Abbreviation: IBS; Irritable Bowel Syndrome

Supplementary Table 2: Stratification of IBS patients based on predominant stool patterns

Timepoint 1				Timepoint 2			
Constipation	Diarrhoea	Mixed	Unspecified	Constipation	Diarrhoea	Mixed	Unspecified
18 (32.7%)	14 (25.5%)	12 (21.8%)	11 (20.0%)	20 (36.4%)	20 (36.4%)	6 (10.9%)	9 (16.4%)

Fifty-five IBS patients were included in the analysis. Data were presented with numbers and ratios in the bracket.

Supplementary Table 3: Dynamics of IBS symptoms over time.

	HA			IBS patients		
	**Timepoint 1**	**Timepoint 2**	**p values**	**Timepoint 1**	**Timepoint 2**	**p values**
**Age (years)**	35.0 (22.5 – 38.4)	35.0 (22.5 – 38.4)	NA	42.0 (26.0 – 52.5)	42.0 (26.0 – 52.5)	NA
**BMI (kg/m²)**	23.3 ± 3.0	23.3 ± 3.0	1.00	22.8 ± 2.8	22.8 ± 2.8	1.00
**IBS-SSS**	60 (30 – 90)	90 (50 - 90)	0.20	140.0 (100.0 – 250.0)	150.0 (110.0 – 225.0)	0.47
**Bristol stool scale**	3 (3 – 4)	3 (3 – 4)	0.61	4 (3 – 6)	4 (3 – 6)	0.63
**Anxiety score**	4.0 (3.0 – 6.0)	3.0 (2.0 – 6.0)	0.71	7.0 (4.0 – 11.5)	7.0 (3.5 – 11.0)	0.60
**Depression score**	2.0 (0.5 – 2.5)	1.0 (0.0 – 2.0)	0.13	2.0 (1.0 – 5.5)	3.0 (1.0 – 6.0)	0.66
**IBS-QoL**	99.3 (98.9 – 100.0)	100 (98.5 – 100.0)	0.60	75.7 (57.4 – 85.3)	77.2 (65.8 – 86.0)	0.10
Dysphoria	100.0 (100.0 – 100.0)	100.0 (100.0 – 100.0)	0.59	78.1 (56.3 – 90.6)	81.3 (65.6 – 92.2)	**0.03**
Interference_with_act	100.0 (100.0 – 100.0)	100.0 (100.0 – 100.0)	1.00	75.0 (60.7 – 85.7)	78.6 (67.9 – 87.5)	0.11
Body_image	100.0 (96.9 – 100.0)	100.0 (100.0 – 100.0)	0.37	75.0 (56.3 – 87.5)	81.3 (62.5 – 90.7)	**0.00**
Health_worry	100.0 (100.0 – 100.0)	100.0 (100.0 – 100.0)	0.82	75.0 (58.3 – 91.7)	75.0 (58.3 – 87.5)	0.59
Food_avoidance	100.0 (100.0 – 100.0)	100.0 (100.0 – 100.0)	0.34	50.0 (33.3 – 75.0)	50.0 (33.3 – 75.0)	0.88
Social_reaction	100.0 (96.9 – 100.0)	100.0 (100.0 – 100.0)	0.28	81.3 (62.5 – 87.5)	81.3 (62.5 – 93.8)	0.86
Sexual	100.0 (100.0 – 100.0)	100.0 (100.0 – 100.0)	1.00	75.0 (50.0 – 100.0)	75.0 (50.0 – 100.0)	0.36
Relationship	100.0 (100.0 – 100.0)	100.0 (100.0 – 100.0)	1.00	83.0 (66.7 – 91.7)	83.0 (75.0 – 91.7)	**0.04**

Data presents mean ± standard deviation or median (interquartile range) when skewed. BMI was tested with an independent sample t-test. Age, IBS-SSS, Bristol stool scale, anxiety score, depression score, IBS-QoL and its subscales (dysphoria, interference with act, body image, health worry, food avoidance, social reaction, sexual and relationship) were tested with Mann-Whitney U test. Abbreviations: BMI, Body Mass Index; IBS-SSS, IBS Symptom Severity Score; IBS-QoL, IBS Quality of Life. NA: not available

Supplementary Table 4: Subjects with HM or LM in HA and IBS patients.

	Timepoint 1				Timepoint 2			
	Total	IBS patients	Healthy adults	p values	Total	IBS patients	Healthy adults	p values
HM n (%)	28 (34.15%)	19 (34.55%)	9 (33.33%)	1.000	26 (31.71%)	18 (32.73%)	8 (29.63%)	0.975

Twenty-seven healthy adults and fifty-five IBS patients are included in the analysis. Subjects were stratified into low-level methanogens (LM) and high-level methanogens (HM) based on the relative abundance of methanogens with a threshold of 0.1%. Data were presented with a number and the ratio in the bracket. The ratio difference between HA and IBS patients was tested with a Pearson's Chi-square test. Abbreviations: HA, healthy adults; IBS, irritable bowel syndrome; HM, High-level Methanogens.

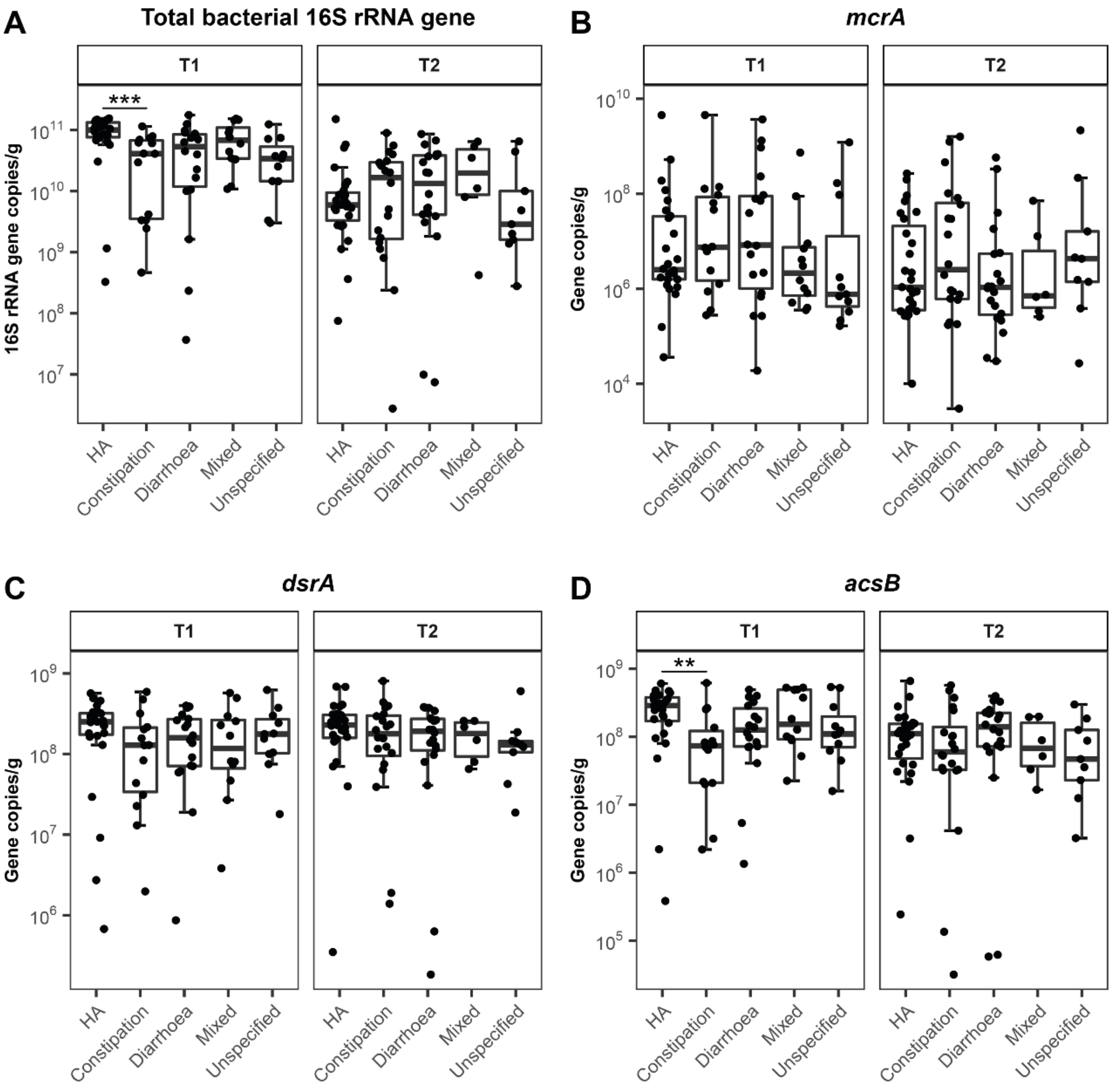

Supplementary Figure 1: Comparison of total bacterial 16S rRNA gene (A) and genes indicative of hydrogenotrophic functional groups (B, *mcrA*, methanogens; C, *dsrA*, sulphate-reducing bacteria; D, *acsB*, acetogens) between IBS subgroups based on predominant stool patterns and healthy adults over time. Values were presented as interquartile with boxplot. Significance between groups was tested with the Mann-Whitney U test. * $p < 0.05$; ** $p < 0.01$.

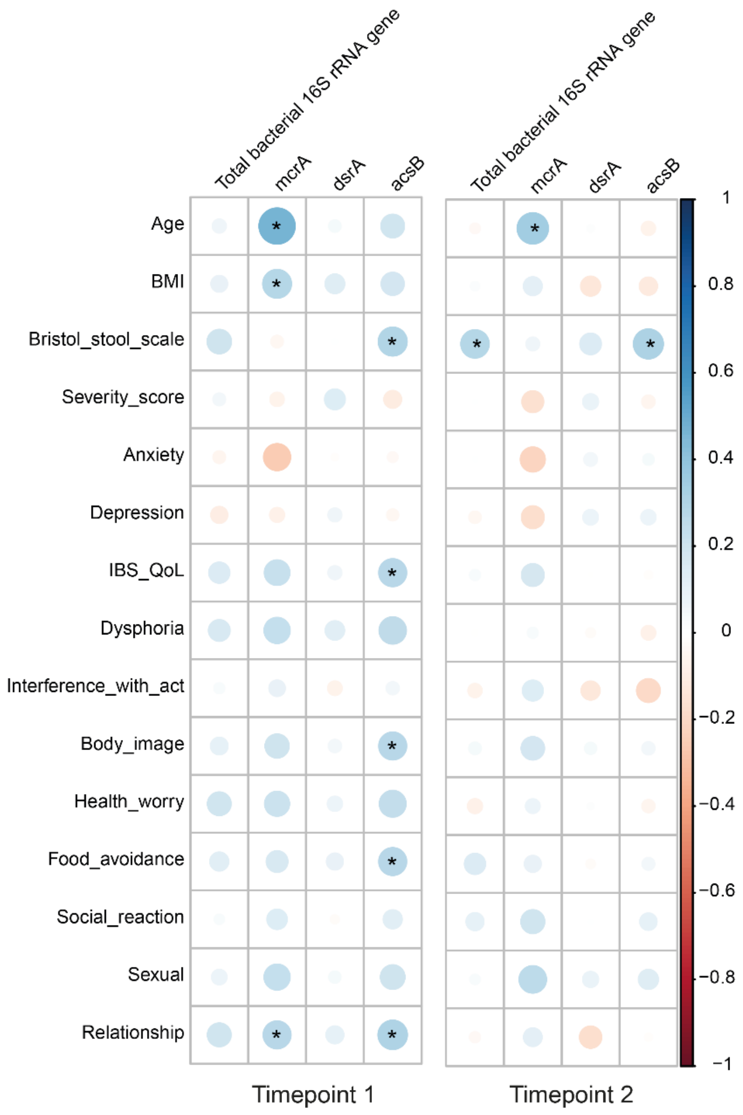

Supplementary Figure 2: Spearman's correlation analyses of total bacterial 16S rRNA gene and hydrogenotrophic functional groups (*mcrA*, methanogens; *dsrA*, sulphate-reducing bacteria; *acsB*, acetogens) with population characteristics over time. This analysis only includes 55 IBS patients. Significant correlations ($p < 0.05$) are indicated with an asterisk. Abbreviations: BMI, body mass index; IBS-QoL, IBS quality of life.

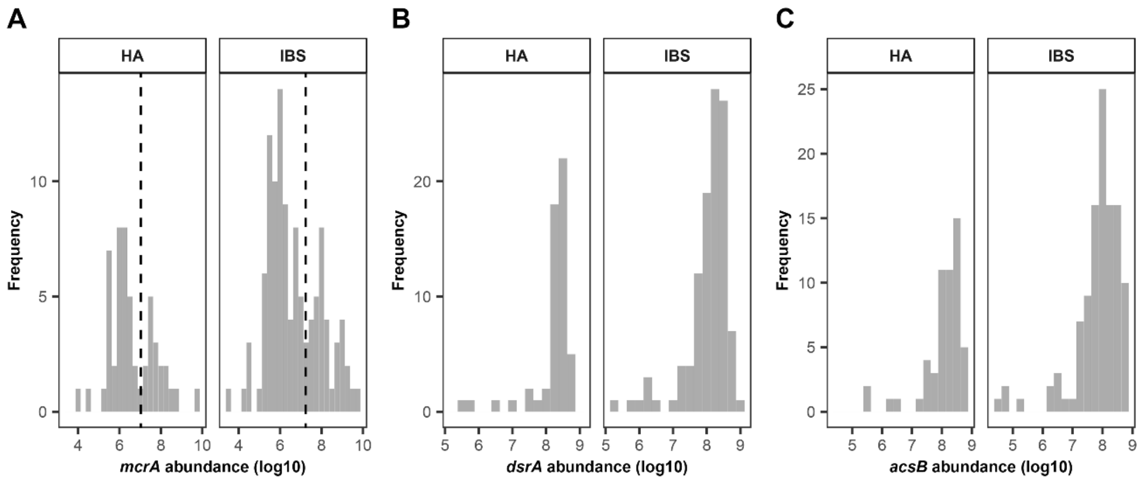

Supplementary Figure 3: Logarithmic abundance distribution of the hydrogenotrophic functional groups (*mcrA*, methanogens; *dsrA*, sulphate-reducing bacteria; *acsB*, acetogens) in HA and IBS patients. (A) *mcrA* showed two distinct peaks, indicating a bimodality of methanogens, which is not found in the logarithmic abundance distributions of (B) *dsrA* and (C) *acsB*.

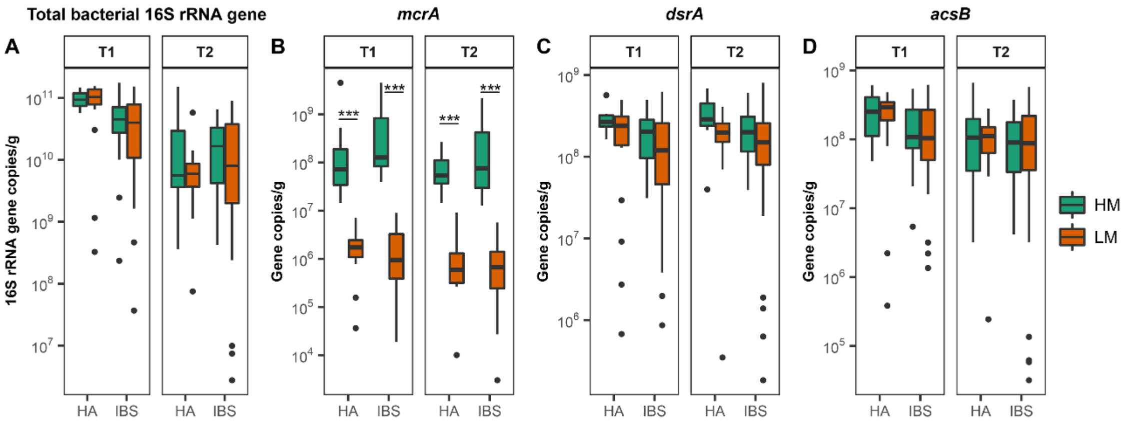

Supplementary Figure 4: Comparison of total bacteria (A) and hydrogenotrophic functional groups (B, *mcrA*, methanogens; C, *dsrA*, sulphate-reducing bacteria; D, *acsB*, acetogens) between HM and LM in HA and IBS patients over time. Data were displayed as interquartile with boxplot. Mann-Whitney U test was used to test the significance. * $p < 0.05$; ** $p < 0.01$, *** $p < 0.001$. HM, High-level methanogens; LM, low-level methanogens. HA, healthy adults.

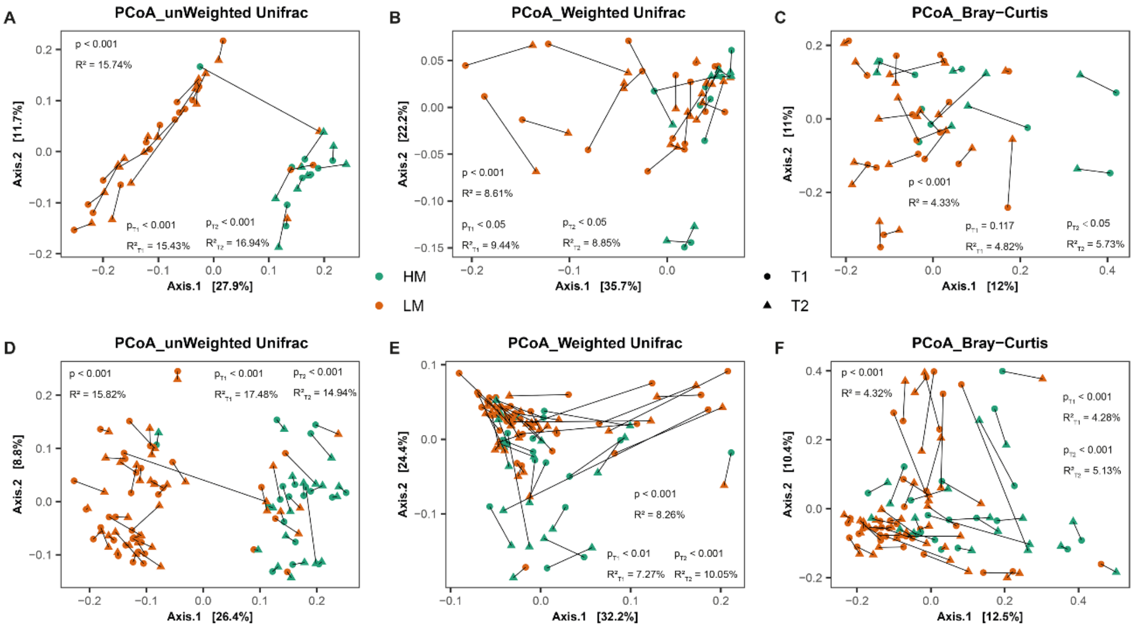

Supplementary Figure 5: PCoA of microbiota composition in HA (A, B, C) and IBS (D, E, F) stratified into HM and LM based on unweighted and weighted Unifrac distances, and Bray-Curtis dissimilarity, respectively. Significant differences between HM and LM in HA and IBS were determined using PERMANOVA with two-timepoint samples included or at each timepoint, respectively. Samples taken at different timepoints are connected by solid lines per subject. * $p < 0.05$; ** $p < 0.01$, *** $p < 0.001$. HM, high-level methanogens; LM, low-level methanogens; HA, healthy adults.

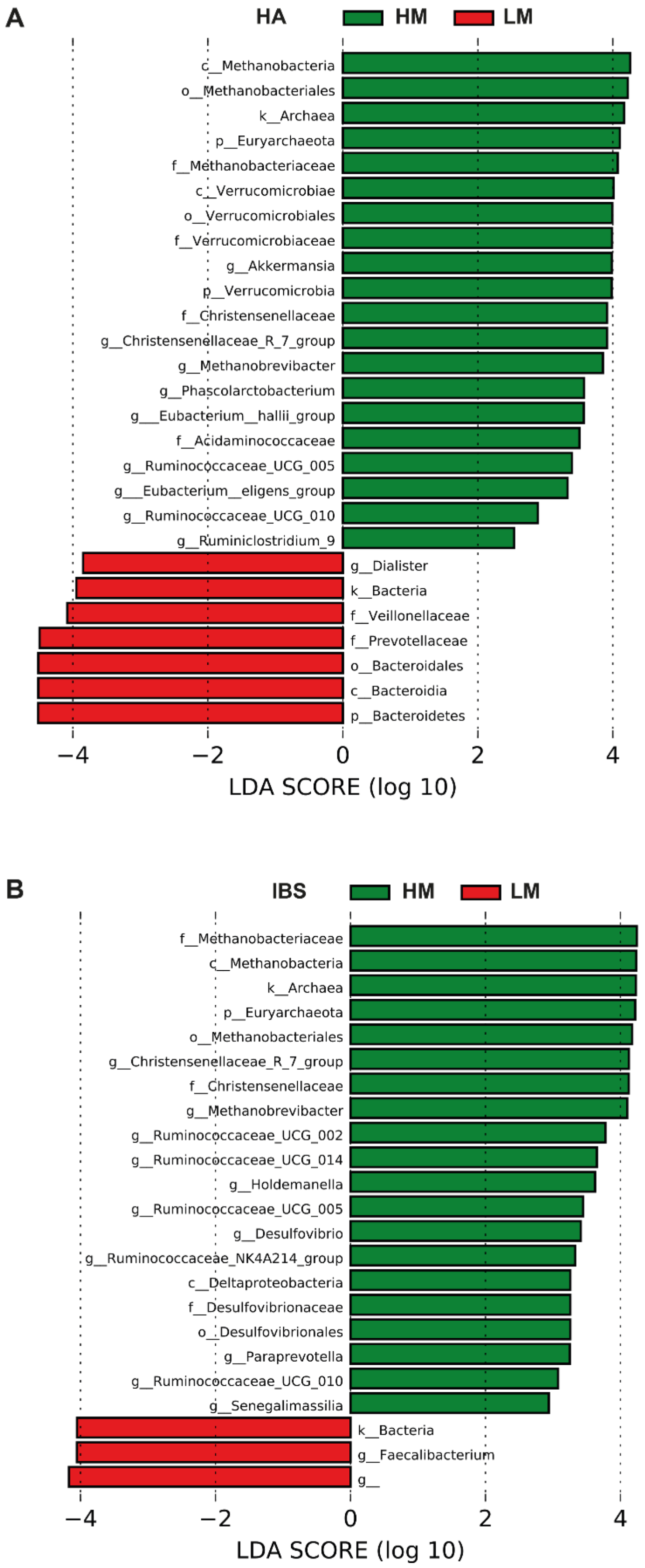

Supplementary Figure 6: Histogram of the linear discriminant analysis (LDA) scores for differentially abundant microbial clades between HM and LM in HA (A) and IBS patients (B). Negative (red bars) LDA scores represent microbial groups over-abundant in LM, while positive (green bars) represent bacterial groups over-abundant in HM. HM, high-level methanogens; LM, low-level methanogens. HA, healthy adults.
